# *De novo* transcriptome sequencing and analysis of *Coccinella septempunctata* L. in non-diapause, diapause and diapause-terminated states to identify diapause-associated genes

**DOI:** 10.1186/s12864-015-2309-3

**Published:** 2015-12-21

**Authors:** Xiaoyang Qi, Lisheng Zhang, Yanhua Han, Xiaoyun Ren, Jian Huang, Hongyin Chen

**Affiliations:** Institute of Plant Protection, Chinese Academy of Agricultural Sciences; Key Laboratory of Integrated Pest Management in Crops, Ministry of Agriculture, Sino-American Biological Control Laboratory, USDA-ARS, Beijing, 100081 China; Key Laboratory of Integrated Pest Management for Fujian-Taiwan Crops, Ministry of Agriculture, College of Plant Protection, Fujian Agriculture and Forestry University, Fuzhou, 350002 China

**Keywords:** *Coccinella septempunctata* L, Diapause, Transcriptome, Fatty acid biosynthesis, Diapause-associated genes

## Abstract

**Background:**

The most common ladybird beetle, *Coccinella septempunctata* L., is an excellent predator of crop pests such as aphids and white flies, and it shows a wide range of adaptability, a large appetite and a high reproductive ability. Diapause research plays an important role in the artificial propagation and shelf-life extension of insect products. Although this lady beetle’s regulatory, physiological and biochemical characteristics in the diapause period are well understood, the molecular mechanism of diapause remains unknown. Therefore, we collected female adults in three different states, i.e., non-diapause, diapause and diapause termination, for transcriptome sequencing.

**Results:**

After transcriptome sequencing using the Illumina HiSeq 2500 platform with pretreatment, a total of 417.6 million clean reads from nine samples were filtered using the program FASTX (version 0.0). Additionally, 106,262 contigs were assembled into 82,820 unigenes with an average length of 921 bp and an N50 of 1,241 bp. All of the unigenes were annotated through BLASTX alignment against the Nr or UniProt database, and 37,872 unigenes were matched. We performed further analysis of these unigenes using the Clusters of Orthologous Groups of proteins (COG), Gene Ontology (GO), and the Kyoto Encyclopedia of Genes and Genomes (KEGG) databases. Through pairwise comparisons of the non-diapause (ND), diapause (D), and diapause-terminated (DT) groups, 3,501 and 1,427 differentially expressed genes (DEGs) were identified between D and ND and between DT and D, respectively. Moreover, 443 of the DEGs were specifically expressed during the diapause period (i.e., DEGs that were expressed at the highest or lowest levels during diapause compared with the other stages). GO function and KEGG pathway enrichment were performed on all DEGs and showed that RNA-directed DNA polymerase activity and fatty acid metabolism were significantly affected. Furthermore, eight specific expressed genes were selected for validation using qRT-PCR. Among these eight genes, seven genes were up-regulated, and one gene was down-regulated; the change trends of the eight genes were the same between the qRT-PCR and RNA-seq analysis results.

**Conclusions:**

In this study, a new method for collecting and identifying diapause insects was described. We generated a vast quantity of transcriptome data from *C. septempunctata* L., providing a resource for gene function research. The diapause-associated genes that we identified establish a foundation for future studies on the molecular mechanisms of diapause.

**Electronic supplementary material:**

The online version of this article (doi:10.1186/s12864-015-2309-3) contains supplementary material, which is available to authorized users.

## Background

Both larval and adult *Coccinella septempunctata* L., the seven-spot ladybird beetle, are excellent predators of multiple aphid species. This species has been widely utilized as a biocontrol agent against various aphids because of its wide range of adaptability, large appetite, high reproductive ability and ease of mass rearing and has yielded numerous ecological and economic benefits [1–3]. The natural enemies of soybean aphids, such as lady beetles (*Harmonia axyridis* and *C. septempunctata* L.), lacewings (*Chrysoperla carnea*), and true bugs (*Orius insidiosus*), appear to play key roles in regulating aphid populations. Among these insects, *C. septempunctata* L. dominates early in the growing season [4–6]. In Europe, experiments on greenhouse crops, such as tomatoes, cucumbers and sweet peppers, have shown that *C. septempunctata* L. is one of the most important species of coccinellids in controlling aphid populations [2]. Pollution from chemical pesticides can be reduced by using lady beetles to control aphids. After late April or early May, the adults that have survived hibernation begin to spawn [7, 8].

Diapause is a genetic phenomenon in which an insect’s growth is almost terminated in response to one or more specific environmental stimuli and can only be reinitiated by another specific stimulus [9–11]; this is a behavior that allows insects to adapt to the environment. Once insects enter into the diapause state, they grow slowly, showing low metabolism or developmental stagnation for a long period in the absence of a specific stimulus to break diapause. Thus, the resistance of the insects is strengthened. Although these characteristics can make artificial propagation more challenging, they are also used to extend the shelf lives of insects. For example, in a study of the physiological characteristics of diapause in the lady beetle *C. septempunctata* L., the insect’s lifespan was significantly extended during diapause to more than 220 days, whereas the typical lifespan of this insect is approximately 63 days [12]. Numerous studies on diapause have elucidated the mechanisms of environmental regulation, particularly the role of the hormonal system in the occurrence and termination of diapause [13–15]. The diapause process can be divided into three phases: pre-diapause, diapause and post-diapause [16]. During the pre-diapause phase, insects perceive the triggering stimulus from the environment but continue to develop. Research on diapause induction in *Bombyx mori* has shown that rearing conditions of 25 °C with a 14-h light/10-h dark cycle can induce diapause [16, 17]. The external direct development of individual form (morphogenesis) begins to gradually slow and eventually ceases during diapause. Finally, during post-diapause, the insects immediately resume development when advantageous environmental conditions arise [18]. Many aspects of the physiological and ecological control of diapause are understood, but the molecular mechanism of diapause remains unclear [19, 20]. In recent decades, diapause research on various insects has been performed [21–23]. Some candidate genes have been found to play significant roles in the process of diapause. For example, a *de novo* transcriptome analysis of the Asian tiger mosquito, *Aedes albopictus* (Skuse), identified several genes, such as receptor for activated C kinase (*rack1*), ecdysone inducible protein L2 (*eip*) and G-protein coupled receptor (*gpcr*), that potentially play complex roles in diapause preparation [22]. In *Culex pipiens*, FOXO (forkhead box protein O) was found to play crucial roles in two characters during diapause: fat hypertrophy and extended lifespan [24]. There has been extensive research on *B. mori* diapause, and the diapause hormone was discovered and named in this species [25–27]. Analogs of this hormone have been found in *Heliothis virescens* and *Helicoverpa armigera* [28, 29], but their function contrasts with that of *B. mori* by ending diapause rather than inducing it. Among ladybugs, comprehensive research on diapause has primarily been conducted on adults of species such as *Harmonia axyridis* [30, 31] and *Exochomus quadripustulatus*. Some ladybugs show geographic diversity in diapause. *Coccinella septempunctata* L. exhibits summer diapause in Greece and winter diapause in central Western Europe [32, 33]. Studies on *C. septempunctata* L. in Beijing have revealed the occurrence of winter diapause, with the highest proportion of diapause occurring under rearing conditions of 18 ± 1 °C with a 10:14 (L:D) h photoperiod [30]. The physiological and ecological controls of diapause are known [6, 34], and the differential expressions of diapause-related proteins in *C. septempunctata* L*.* have been studied in our laboratory [35], whereas the molecular bases of diapause remain unclear. Recently, next-generation sequencing technology has greatly promoted the development of insect transcriptomics, especially for insects without reference genome sequences [36–38].

Next-generation sequencing technology (NGS), also known as high-throughput sequencing technology, collects massive amounts of sequence data in a single run [39, 40]. The emergence of NGS has greatly promoted the study of insect transcriptomes. To date, the transcriptomes of 68 species from 7 insect orders have been sequenced [41–43], and the transcriptome and expression profile data of some lady beetles such as *Cryptolaemus montrouzieri* Mulsant and *Propylaea japonica* (Thunberg) have been analyzed [44, 45]. For organisms without reference genomes, *de novo* transcriptome sequencing can not only produce genetic information for the species but also be used to predict possible non-coding RNA [46, 47]. Transcriptome sequencing of insect samples obtained under different growing conditions or from different habitats or species not only generates functional gene information and reveals differentially expressed genes but also provides the abundance of expressed transcripts, the loci where transcription occurs, transcript SNPs and other important information [48–51]. We conducted research on diapause in *C. septempunctata* L. based on the results of other insect studies [52].

In this study, we used RNA sequencing to identify differentially expressed genes related to diapause by comparing non-diapause, diapause, and diapause-terminated groups; a total of 461 diapause-associated genes (DGs) were identified (i.e., DEGs that were expressed at the highest or lowest levels during diapause compared with the other stages). To verify the precision of the transcriptome results, we selected 8 DGs via quantitative real-time PCR (qRT-PCR): sorbitol dehydrogenase (*sdh*), glycogen phosphorylase (*gp*), toll-like receptor 2 (*tlr2*), serine protease (*sp*), juvenile hormone epoxide hydrolase (*jheh*), acetyl-CoA carboxylase (*acc*), trehalase (*tre*) and an uncharacterized protein, *LOC100142139*. These DGs were specifically expressed in diapause and play important roles in energetic metabolism, immune defense, hormone regulation, and other functions. We discuss the molecular functions and regulatory mechanism of the DGs relative to those from other insects in this manuscript. Some significant pathways, such as the insulin/FOXO signaling pathway and the cell cycle, were also analyzed. The transcriptome results for *C. septempunctata* L. enrich the available information on insect diapause and provide an improved method for collecting and identifying insects in diapause.

## Results and discussion

### Results

#### Experimental insects in diapause

**Fig. 1:**
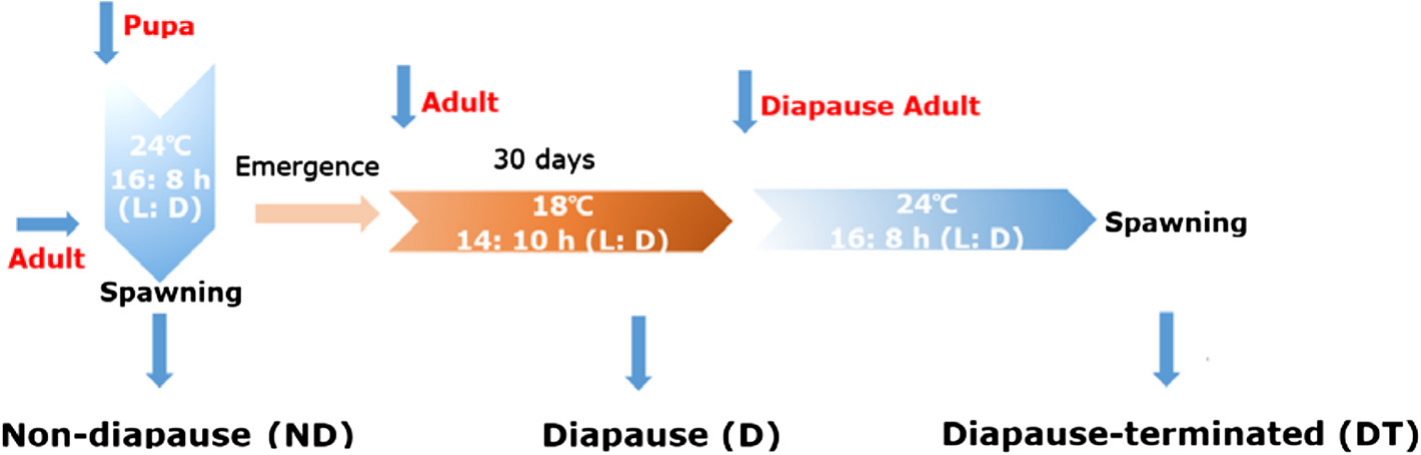
Schema of sampling for transcriptome sequencing. All the insects are reared at 24 °C with long-day from egg to pupa and emergence for sampling

In adult insects, the most obvious and easily recorded feature of diapause is that reproduction is inhibited. Current research indicates that adult diapause can be reflected by specific indices, such as the time of pre-spawning, the developmental process of the ovary and ova, reproductive activity, and body surface features [53, 54]. In Hokkaido, Japan, it was found that the ovarian development of adult *C. septempunctata* L. females nearly ceases and that the eggs in the ovary are immature. Moreover, egg cell development is inhibited, and the developmental stages usually do not exceed the period of yolk formation [55]. A study from our laboratory examining the preliminary ecology of *C. septempunctata* L. in Beijing, China, showed that diapause occurred in adult *C. septempunctata* L. We further found that the adult diapause rate was highest when the temperature and photoperiod conditions were 18 °C and 10:14 h (L:D), respectively, and that the female ovary was transparent, ovarioles showing yolk deposition were absent, and the egg tubules were not obviously differentiated under these conditions [56]. Therefore, in the present study, all of the experimental insects in diapause were reared under a normal developmental temperature of 24 °C, a photoperiod of 16:8 h (L: D) and an RH of 70 ± 10 % until emergence (Fig. 1). After emergence, the insects were immediately transferred to another artificial climate box with a temperature and photoperiod of 18 °C and 10:14 h (L: D), respectively, and 2 female and 2 male insects were bred in one plastic cup for 30 days. Then, one insect was randomly selected for dissection to observe ovary development; the other female adult insect was identified as a diapausing individual because the ovary was undeveloped.

### Illumina sequencing and *de novo* assembly

**Fig. 2:**
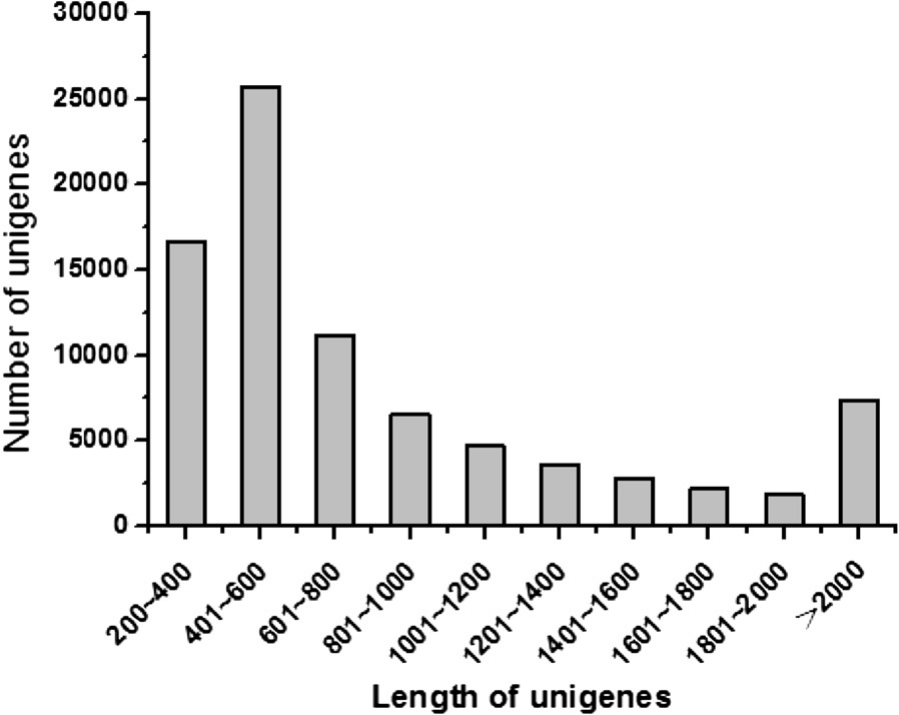
Length distribution of unigenes. A total of 82,820 unigenes were assembled. The x-axis represents the length, and the y-axis represents the number of unigenes

According to the data obtained through sequencing using the Illumina HiSeq 2500 platform, we performed routine transcriptome analysis on adult female *C. septempunctata* L. in different developmental stages: non-diapause, diapause, and diapause-terminated. With three biological replicates of three samples, the 9 libraries yielded 453,401,612 raw reads, comprising 154,790,202, 145,249,536 and 153,361,874 reads for the non-diapause (ND), diapause (D) and diapause-terminated (DT) groups, respectively (Table 1). After adaptor trimming and quality filtering, a total of 417,628,298 clean reads were filtered, and 106,262 contigs were assembled using CLC Genomics Workbench (version 6.0.4), with a mean length of 713 bp (Table 1). After the assembly of one or more contigs, 82,820 unigenes were generated with an average length of 921 bp (Additional file 1). The lengths of the assembled unigenes were primarily in the range of 200–1,000 bp (approximately 72.67 % of the total unigenes were in this range), with most unigenes falling between 401 bp and 600 bp and some exceeding 2000 bp (Fig. 2). The results of open reading frame (ORF) prediction demonstrated that 38,488 of 82,820 unigenes contained one or more ORFs.

**Table 1:** Summary statistics from Illumina sequencing of the *C. septempunctata* L. transcriptome

Sequencing	
Total number of reads	453,401,612
Number of clean reads	417,628,298
Number of contigs	106,262
Total length of contigs (bp)	75,724,113
Contig N50	929
Average length of contigs (bp)	713
Number of primary unigenes	84,291
Number of final unigenes	82,820
Total length of final unigenes (bp)	76,279,917
Final unigene N50	1,241
Average length of final unigene (bp)	921
GC percentage (%)	42. 4
N percentage (%)	1. 2
Annotation	
Unigene annotations against Nr	35,115
Unigene annotations against UniProt	37,415
Total number of Unigene annotations	37,872
Functional classifying and pathway mapping	
Unigene annotations against KEGG	18,286
Unigene annotations against GO	112,402
Unigene annotations against COG	100,308

### Annotation of unigenes

**Fig. 3:**
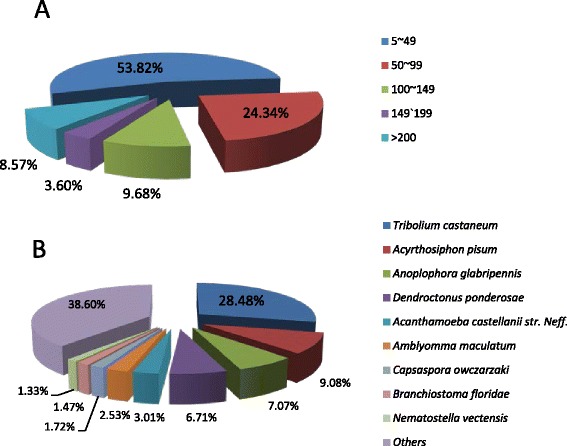
Homology searches of Illumina sequences against the UniProt and Nr database. **a** E-value distribution. The filtering conditions for the unigenes matched against the UniProt and the Nr databases were set to a cut-off value of 1e-5. The range of 5–10 represents 1e-5-1e-10. **b** Species distribution of BLASTX hits. The species were determined based on the highest score in the Bx results

When the unigenes were annotated according to the UniProt and Nr databases using BLASTX, 37,872 of 82,820 unigenes were annotated (approximately 45.70 % of all of the unigenes), and 37,415 contigs were filtered with a cutoff e-value of 1e-5, among which 46.2 % of the homologous sequences showed values below 1e-50 (Fig. 3a). Because the genome sequence of *C. septempunctata* L. has not been reported, sequence alignment of the experimental unigenes was performed using the known genomes of other species. In the species distribution, the top matches were obtained for *Tribolium castaneum*, with 10,655 sequences (28.5 %), followed by *Acyrthosiphon pisum* (9.03 %), *Anoplophora glabripennis* (7.07 %), *Dendroctonus ponderosae* (6.71 %), *Acanthamoeba castellanii* str. Neff*.* (3.01 %) and other species (45.68 %) (Fig. 3b).

**Fig. 4:**
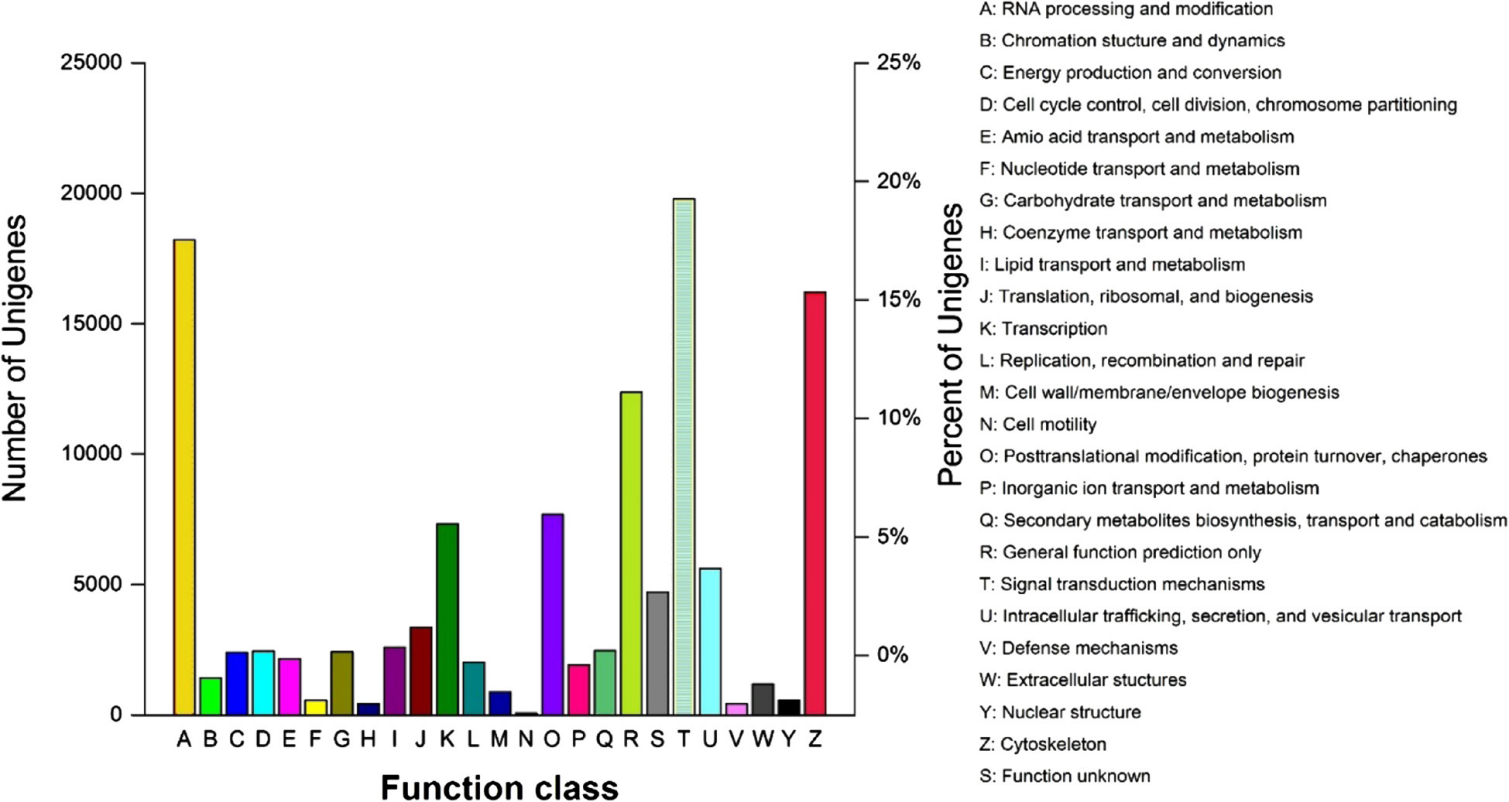
Clusters of Orthologous Groups (COG) functional categories in the *C. septempunctata* L*.* transcriptome. A total of 32,350 unigenes were annotated into 25 categories. The left y-axis represented number of unigenes, and right y-axis represented percent of unigenes

**Fig. 5:**
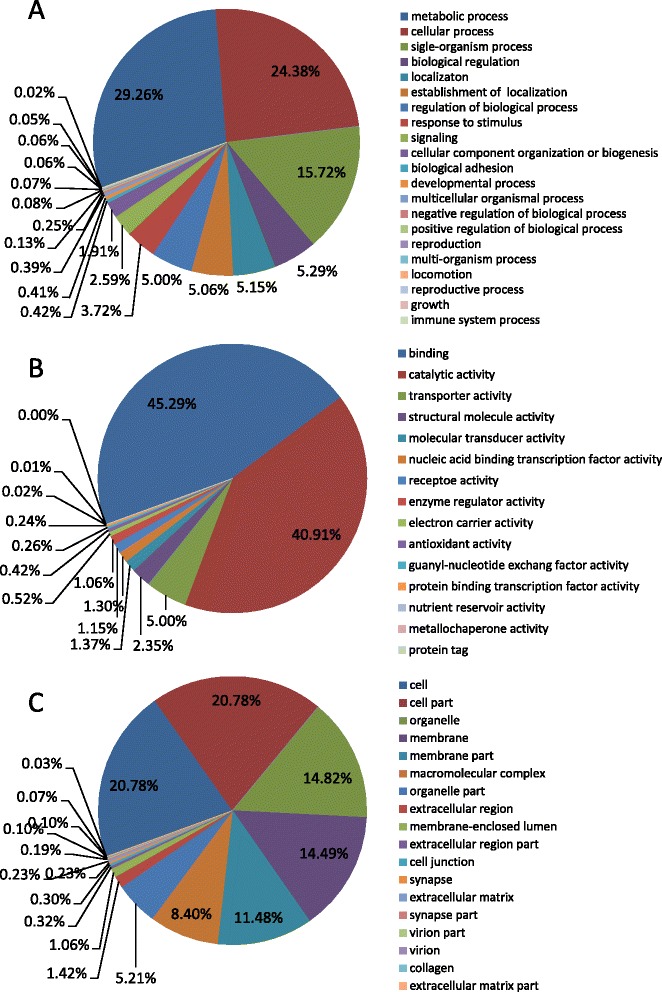
Gene ontology (GO) classifications for the *C. s*eptempunctata L. transcriptome. In the graph, **a**, **b**, and **c** represent biological processes, molecular functions, and cellular components, respectively

To further analyze the integrity of the libraries and the effectiveness of the annotation process, COG functional classification was performed on the unigene alignment with the CDD database [57] using the rpstBlastn program; 32,350 unigenes were annotated to 25 COG categories (Fig. 4). The largest group in the cluster was “signal transduction mechanisms”, with 19,785 unigenes; followed by “RNA processing and modification”, with 18,209 unigenes; “cytoskeleton”, with 16,201 unigenes; “general function prediction only”, with 12,382 unigenes; and “posttranslational modification, protein turnover, chaperones”, with 7,688 unigenes. These results demonstrated that signaling transduction mechanisms were the most important in adult female *C. septempunctata* L.. Gene Ontology (GO) is an international standardization of the gene functional classification system. Improvement of the biological annotation of genes contributed to the development of GO classification, and the three-class system (biological processes, molecular functions, and cellular components) plays a key role in the bioinformatics annotation process. In this experiment, biological processes, molecular functions, and cellular components were associated with 20,990 unigenes, 22,866 unigenes, and 9,961 unigenes, respectively (Additional file 2). Among biological processes, metabolic processes (29.26 %) and cellular processes (24.38 %) were the most abundant groups, whereas immune system processes (0.02 %), growth (0.05 %) and locomotion (0.06 %) were the least abundant groups (Fig. 5a). In terms of molecular functions, binding (45.29 %) and catalytic activity (40.91 %) accounted for the majority of unigenes in the unigene classification, whereas protein tags (near 0.00 %), metallochaperone activity (0.01 %), and nutrient reservoir activity (0.02 %) were the least abundant categories (Fig. 5b). Among the cellular components, the cell (20.78 %) and cell part (20.78 %) categories were the most abundant, followed by the organelle (14.82 %) and membrane (14.49 %) categories, whereas extracellular matrix (0.03 %) and collagen (0.07 %) were the least abundant categories (Fig. 5c).

When the biologically complex activity of genes was examined using KEGG, 11,949 unigenes were mapped to 308 pathways based on matching with the KAAS (KEGG Automatic Annotation Server). Among these pathways, metabolic pathways, biosynthesis of secondary metabolites, and microbial metabolism in diverse environments were the most represented, with 3,800 unigenes, 1,600 unigenes, 1,217 unigenes, respectively. We identified the areas of interest to further analyze these annotations, providing a valuable resource for elucidating functional genes in adult female *C. septempunctata* L..

### Analysis of gene expression profiles

**Fig. 6:**
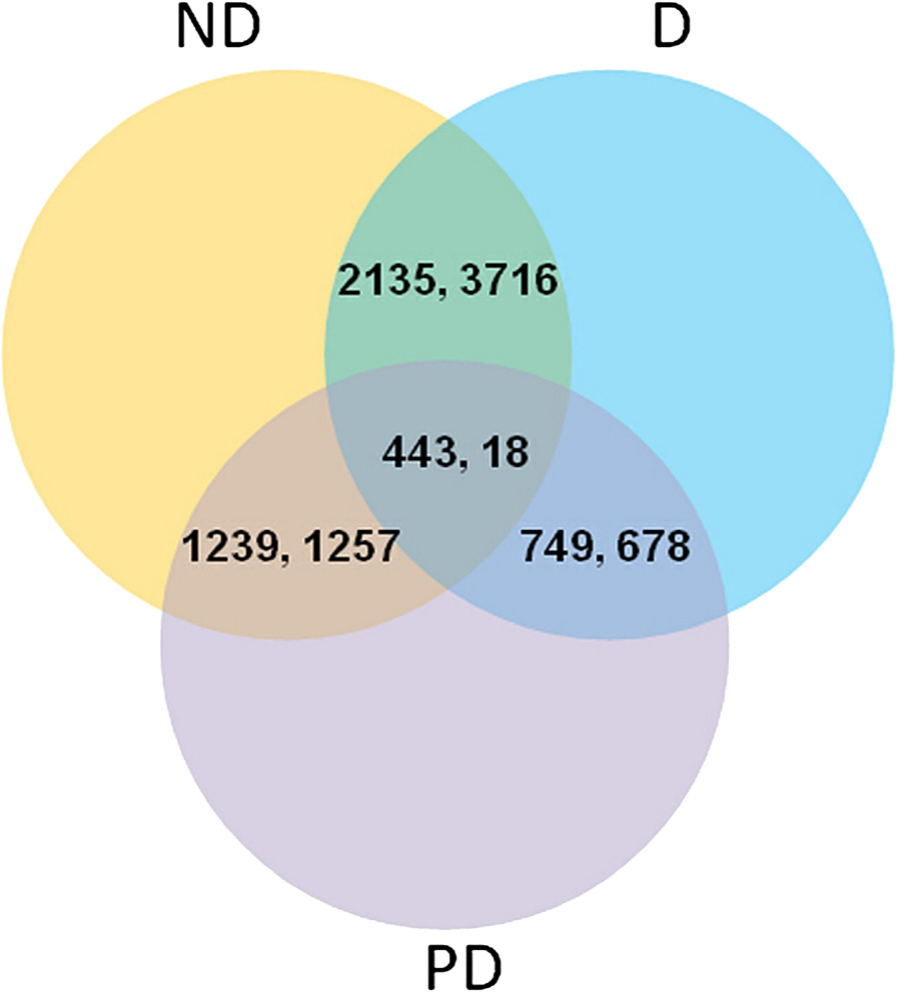
Summary of differentially expressed genes in three libraries identified through pairwise comparisons. The overlapping areas represented the genes we filtered by comparing two or three groups. In each pair of values, the first pair represents the number of up-regulated unigenes, and the second pair represents the number of down-regulated unigenes. ND: non-diapause, D: diapause, DT: diapause-terminated

**Fig. 7:**
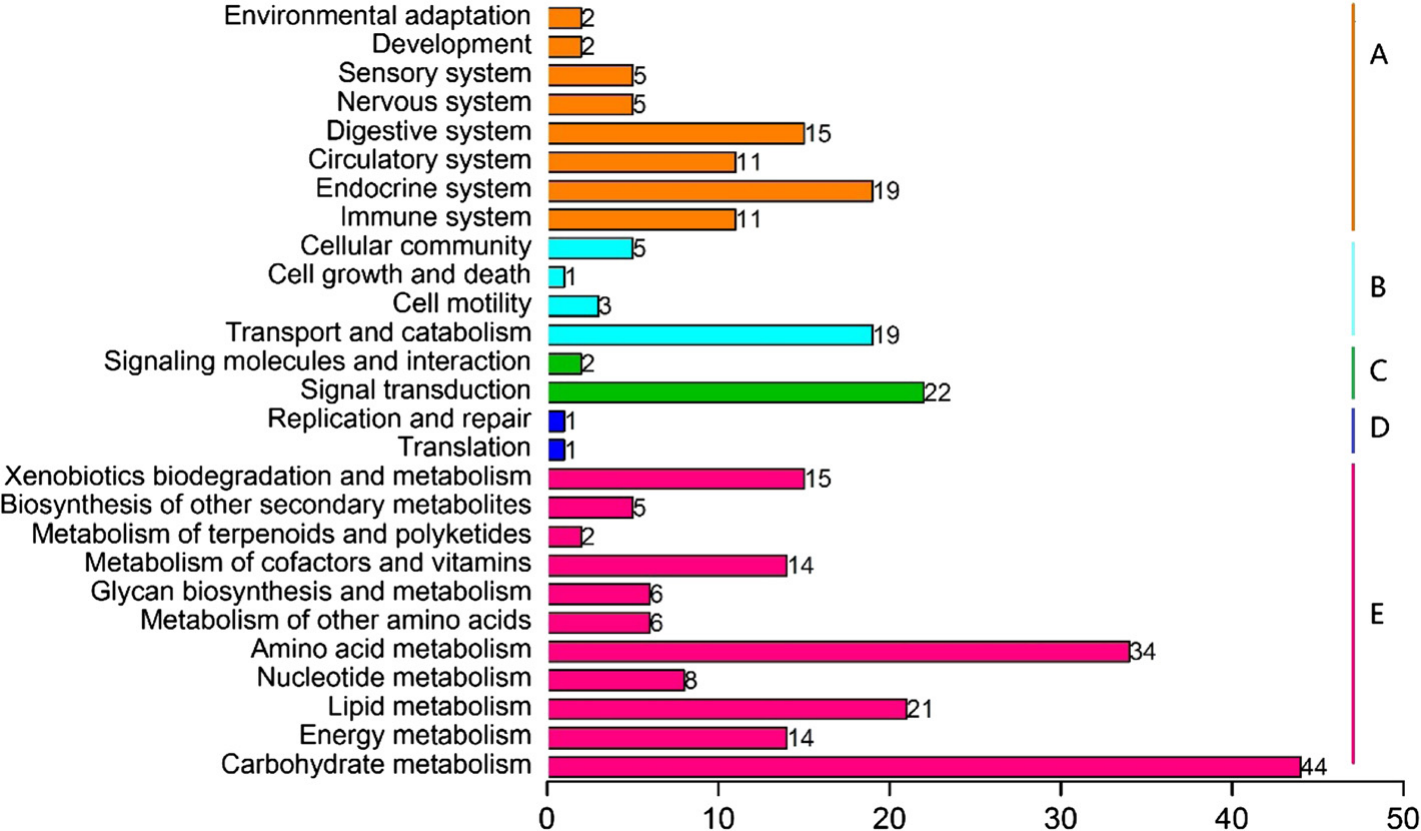
KEGG Orthology (KO) classifications of up-regulated differentially expressed genes (DEGs). 443 DEGs were mapped to five categories; *a*: Organismal Systems; *b*: Cellular Processes; *c*: Environmental Information Processing; *d*: Genetic Information Processing; *e*: Metabolism

To identify significant expression changes in genes, we conducted a differential expression analysis of unigene expression through pairwise comparisons of the three different conditions, non-diapause, diapause, and diapause terminated, and the DEGs were filtered using an FDR (false discovery rate) ≤0.05 and a fold change ≥2. Because of the slow pace at which physiological activities and growth occur during the diapause stage, most genes were silent; however, the expression of some genes remained up-regulated. When ND and D were compared, 2,125 up- and 1,376 down-regulated unigenes were revealed, and 749 up- and 678 down-regulated unigenes were detected between D and DT. To filter the DEGs related to diapause, we examined the unigenes showing higher or lower expression levels in D compared with ND and DT. A total of 443 up- and 18 down-regulated unigenes in the diapause phase were identified based on the annotation results (Fig. 6). Additionally, the up-regulated DEGs were classified into five categories with 27 terms via KEGG Orthology (KO) (Fig. 7).

### Functional enrichment analysis of differentially expressed genes

To understand the functions of the differentially expressed genes, we compared the GO terms associated with the three different treatments after mapping all of the differentially expressed genes to the GO database. In D/ND and DT/D, 793 GO terms (including 289 biological processes, 421 molecular functions, and 83 cellular components) and 459 GO terms (including 173 biological processes, 240 molecular functions, 46 cellular components), respectively, classified all of the differentially expressed genes. We selected 186 terms as significantly enriched terms for D/ND and 155 such terms for DT/D to further comprehend the function of the differentially expressed genes with a cutoff FDR of 0.05. Moreover, we performed an analysis of significantly enriched pathways using a hypergeometric test, comparing the whole genomic background to detect the significantly enriched pathways of the differentially expressed genes. The results showed that 1,174 differentially expressed unigenes and 350 differentially expressed unigenes mapped with 261 and 233 pathways on D/ND and DT/D, respectively. Among these, 74 pathways and 19 pathways were significantly enriched with the threshold set to an FDR ≤0.05 (Table 2 and Additional file 3) (the threshold for D/ND was decreased to FDR ≤0.001 due to the excess number of significantly enriched pathways; the remaining pathways are listed in Additional file 3). A comprehensive comparative analysis of these significantly enriched pathways showed that fatty acid metabolism was the most significantly enriched pathway.

**Table 2:** significantly enriched KEGG pathways in D vs. ND and DT vs. D

Comparison	KEGG pathway	Up genes	Down genes
D *vs* ND	Fatty acid metabolism	55	8
	Biosynthesis of secondary metabolites	174	74
	Citrate cycle (TCA cycle)	41	7
	Carbon metabolism	78	27
	Pyruvate metabolism	40	13
	Ribosome biogenesis in eukaryotes	0	2
	Glycolysis / Gluconeogenesis	44	18
	Fatty acid biosynthesis	33	5
	Galactose metabolism	27	8
	Cardiac muscle contraction	36	0
	Biosynthesis of amino acids	60	28
	Starch and sucrose metabolism	41	22
	Oxidative phosphorylation	64	8
	Lysosome	48	9
	Carbon fixation in photosynthetic organisms	24	18
	beta-Alanine metabolism	22	8
	Ribosome	127	36
	Carbon fixation pathways in prokaryotes	22	4
	Propanoate metabolism	23	0
	Fatty acid degradation	28	5
	Fructose and mannose metabolism	32	13
	Signaling pathways regulating pluripotency of stem cells	0	2
	One carbon pool by folate	26	2
	Drug metabolism - other enzymes	26	2
	Spliceosome	4	14
	Meiosis - yeast	1	1
	Cell cycle	0	7
	Phagosome	34	17
	Aminoacyl-tRNA biosynthesis	8	4
	Aflatoxin biosynthesis	10	0
	Nucleotide excision repair	0	1
	Ras signaling pathway	3	1
	AMPK signaling pathway	40	10
	Epstein-Barr virus infection	4	12
	Arginine and proline metabolism	25	9
TD *vs* D	Cardiac muscle contraction	0	14
	Drug metabolism - other enzymes	2	11
	Fatty acid metabolism	2	18
	Carbon fixation pathways in prokaryotes	0	11
	Spliceosome	1	0
	Ribosome	3	9
	MicroRNAs in cancer	12	6
	Citrate cycle (TCA cycle)	1	14
	Styrene degradation	0	4
	Vitamin digestion and absorption	2	3
	Tryptophan metabolism	1	9
	Tyrosine metabolism	1	8
	Cutin, suberine and wax biosynthesis	1	5
	One carbon pool by folate	1	9
	Photosynthesis - antenna proteins	0	9
	Fatty acid degradation	1	10
	Insulin signaling pathway	8	10
	Novobiocin biosynthesis	0	2
	Adrenergic signaling in cardiomyocytes	0	13

Note: In D vs. ND, all FDR ≤ 0.001; and in TD vs. D, all FDR ≤ 0.05

### Fatty acid biosynthesis

Adults of *C. septempunctata* L. feed during diapause; however, their food intake is 80 % lower than that of non-diapause adults [36]. Therefore, the accumulation of nutrients is crucial before entry into diapause. Lipids store metabolic reserves of macronutrients, and triacylglycerides are the primary form of storage lipids in insects [58]. One remarkable physiological characteristic of *C. septempunctata* L. in diapause is fat hypertrophy. Furthermore, fatty acids are one of the substrates of triacylglyceride synthesis. In comparisons of the KEGG enrichment pathways identified between D/ND and DT/D, we found that there were many more up-regulated genes than down-regulated genes. Among these pathways, all of the 9 DEGs in the KEGG enrichment pathways identified between DT/D were down-regulated; therefore, we filtered the 9 unigenes (7 fatty acid synthase and 2 long-chain acyl-CoA synthetases) with a fold change ≥2 and an FDR ≤0.05, which were up-regulated at diapause and down-regulated at diapause termination (Additional file 4).

### Validation of unigenes using qPCR

**Fig. 8:**
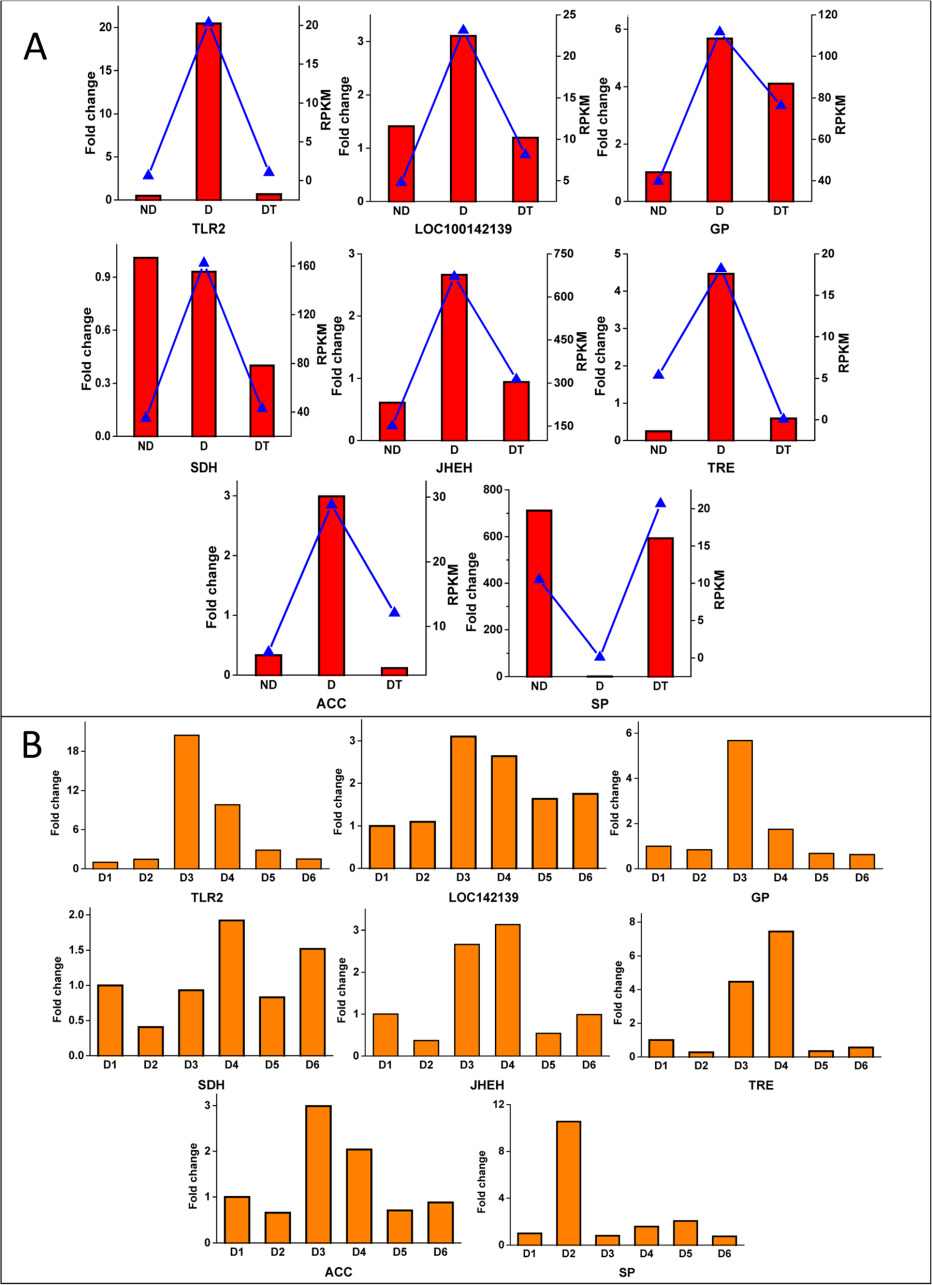
Validation of candidate differentially expressed genes. **a** Expression of differentially expressed genes compared between the qRT-PCR and the RNA-seq results in the three stages: non-diapause, diapause and diapause-terminated. The qRT-PCR results are shown in red, and the values are shown on the left y-axis; the RNA-seq results are shown with blue lines, and the values correspond to the right y-axis. **b** qRT-PCR validation of 6 treatments at diapause stages D1, D2, D3, D4, D5, and D6,correspondingto 10, 20, 30, 40, 50, and 60 days, respectively

The 8 DEGs sorbitol dehydrogenase (*sdh*), glycogen phosphorylase (*gp*), toll-like receptor 2 (*tlr2*), serine protease (*sp*), juvenile hormone epoxide hydrolase (*jheh*), acetyl-CoA carboxylase (*acc*), trehalase (*tre*) and the uncharacterized protein *LOC100142139* were validated through quantitative real-time PCR (Additional file 5). The results of all gene validations are shown in Fig. 8. In Fig. 8a, the RPKM values for RNA-seq and the fold changes detected through qRT-PCR are shown in a single chart to facilitate comparison of the differences. Additionally, the change trends of the 8 DEGs at 6 diapause periods (D1, D2, D3, D4, D5 and D6) are shown in Fig. 8b. Peak expression was observed during diapause at 30 or 40 days except for SP. In general, the results of qRT-PCR verified the reliability of the *de novo* transcriptome sequencing of *C. s*eptempunctata L. as the validation results were consistent with transcriptome sequencing regarding the expression level at the three stages.

## Discussion

Insect diapause may be triggered by various hostile environmental conditions, sudden changes in temperature or photoperiod, or shortage of food [11, 19]. Studies of the regulation of diapause have demonstrated that a number of genes are silent or uniquely expressed during the diapause period; for example, in *Sarcophaga crassipalpis*, *hsp*70 (heat shock protein) is uniquely expressed while *hsp*90 is lowly expressed at the diapause stage [59, 60]. These genes have been reported to be involved in the change of biochemical pathways, changes in metabolic products, energy production, and stress-tolerance enhancement. Usually, diapause is restricted to a specific stage, such as the larva or adult stage [19]. Although the characteristics and functions of a few diapause-associated genes vary with diapause stage, our study on the regulation diapause-associated genes in *C. septempunctata* L. might be useful for future studies of the molecular mechanisms in other species. However, because of the lack of information on gene functions and other characteristics, approximately 54.3 % unigenes were not annotated to any functional genes in our study; their functions will be addressed in our subsequent studies. The present study had some limitations. Although a low temperature is the key condition for inducing artificial diapause in ladybirds, differences in temperature among the diapause groups might have induced variation in developmental kinetics, which subsequently might have induced variation in gene expression. In addition, diapause-terminated females are 10 to 20 days older than are diapausing females because of the time required to break diapause and spawn following the presentation of specific external environmental stimuli: 10 to 20 days are required following the changes in temperature and photoperiod. Diapause is a process in which the stages of diapause, i.e., pre-diapause, diapause, and post-diapause, and the breaking of diapause occur chronologically; therefore, the studied diapause-terminated females were older than the diapausing females. The potential difference in gene expression due to this age difference cannot be eliminated from our experiment. To investigate whether the filtered genes are diapause-associated genes or are genes associated with other, age-related processes, more advanced biological techniques for functional analysis are needed.

### Diapause-associated genes identified by comparing among the three stages

We filtered 461 DEGs by comparing the D group to the ND and DT groups. Among these DEGs, 443 were up-regulated in diapause and were mostly involved in carbohydrate metabolism, amino acid metabolism, signal transduction, and lipid metabolism; carbohydrate metabolism was most commonly represented, with 44 DEGs involved (Fig. 7). Many genes were involved in different pathways. Seven diapause-associated genes with high differentially expression values were *tlr2, jhe, jheh, sdh, tre, fah* and *sp*. With the exception of *sp*, all of these genes were up-regulated during diapause. Some of these genes have been not reported previously in insect diapause. For example, Toll-like receptors (TLRs) are key components of innate immunity and the activation of adaptive immunity [61]. *Contig 19695* was predicted to encode TLR2, and the expression level in D was up-regulated by more than 32-fold and 19-fold compared with ND and DT, respectively. TLR2 is a membrane protein involved in several signal transduction pathways, such as the Toll-like receptor signaling pathway, and plays an important role in the immune surveillance of various organs in mammals and fish [62]. Fumarylacetoacetase (FAH, *Contig 14637* and Additional file 5) is the final and rate-limiting enzyme in tyrosine degradation. Tyrosine is a substrate for melanogenesis through tyrosine metabolism. In melanin cells, the conversion of tyrosine into dopa are catalyzed by tyrosinase, after which oxidation generates dopaquinone to enter the pathway of melanogenesis [63]. Further research on the function of FAH in insect diapause is needed.

Studies of diapause in other insects suggest that it is regulated and controlled by hormones [19]. *jheh* (*Contig 105*) and juvenile hormone esterase (*jhe*, *Contig 7594* and Additional file 5), the enzymes responsible for juvenile hormone (JH) metabolism, regulate the titer of JH in the hemolymph. Based on the results of the transcriptome analysis, we found that the expression value of *jheh* increased during diapause and decreased after diapause. Most studies of JH and diapause show that the absence of JH is the main factor controlling diapause without the corpora cardiaca-corpora allata (CC-CA) complex in insects [64]. Furthermore, the levels of JH III in the hemolymph of pre-diapause and diapause adults were revealed to be low compared to normal developmental adults under long-day photoperiod using JH titer determinations, and the activity correlation between JHE and JH was found to be negative in the Colorado potato beetle, *Leptinotarsa decemlineata* [65–67]. The up-regulation of JHEH and JHE may be due to the decline in JH *in vivo* during diapause in the adult blow fly *Protophormia terraenovae* [68]. We found that *sdh* (*Contig 2214*) expression in the D group was 4 times higher than that in ND and DT. In *Wyeomyia smithii*, the expression of SDH during larval diapause is high, and after diapause, its expression is reduced. Conversely, SDH is expressed at low levels in the silkworm *B. mori* during egg diapause and has been shown to play a key role in diapause termination [69]. These results indicate that changes in SDH expression are not consistent among different diapause types. TRE (*Contig16915*), a glycoside hydrolase enzyme, catalyzes the conversion of trehalose into glucose. In *C. septempunctata* L., it is expressed more during diapause than in the two other phases. Many reports have indicated that in *B. mori*, trehalase activity in the ovaries is controlled by a diapause hormone during the diapause stage and that this hormone enhances the transcriptional activity of the trehalase gene in developing ovaries [70, 71]. According to the results of the DEGs, the number of genes that were down-regulated during diapause was significantly lower than the number of up-regulated genes in *C. septempunctata* L.. SP (*Contig 1935*), an important hydrolytic and digestive enzyme in insects, was found to be down-regulated during diapause in *C. septempunctata* L., with the RPKM value during diapause being 100 times lower than that during the non-diapause stage and 500 times lower than that in the diapause-terminated stage. Trypsin and chymotrypsin-like serine protease, genes encoding two blood-digestive enzymes in the class of serine proteases, were down-regulated in diapause-destined females in the mosquito *C. pipiens*, suggesting that the expression of genes associated with the digestion of a blood meal has been shut down [72].

### Diapause-associated pathways

In *B. mori* and *S. crassipalpis*, the cell cycle is arrested during diapause [73]. Moreover, studies on cell cycle regulatory genes in the brain of *S. crassipalpis* during diapause have shown that cell nuclear antigen (*PCNA*) is expressed at very low levels. In contrast, during the non-diapause or diapause-terminated stage, the expression of *PCNA* is high. Based on KEGG enrichment analysis of D vs. ND and DT vs. D, we showed that all seven DEGs were down-regulated in D compared with ND and that no DEGs were up-regulated in D. Furthermore, a total of 15 DEGs were annotated in the comparison of DT and D, including wee1-like protein kinase [EC:2.7.11.1], polo-like kinase 1 [EC:2.7.11.21], DNA replication licensing factor MCM6 [EC:3.6.4.12], cyclin A/B (CYCA/B), S-phase kinase-associated protein 1/2 (SKP1/2), cell division cycle 20 (CDY20), anaphase-promoting complex subunit 1 (APC1), and origin recognition complex subunit 5 (ORC5), all of which were up-regulated in DT (Additional file 4). Thus, cell cycle arrest might be a hallmark of ladybug diapause, although the specific phase was undefined in *C. septempunctata* L. The insulin signaling pathway has been the subject of much research in insect diapause. Studies on insulin signaling might be a unifying direction for diapause research. Work examining diapause in *Drosophila melanogaster* and the mosquito *C. pipiens* revealed that the insulin signaling pathway is involved in the regulation of the diapause phenotype. More recent studies have discussed the regulatory phenotype of diapause, including lifespan extension, stagnated reproduction, suppressed metabolism, and enhanced stress tolerance and fat accumulation. FOXO, a forkhead transcription regulatory factor, plays a key role in growth and development, metabolism, aging and immunity. For example, in *Drosophila*, activated dFOXO is involved in G1 cell cycle arrest [74]. In *C. pipiens*, research on the regulation of insulin signaling and FOXO indicates that under normal developmental conditions, insulin signaling prompts the synthesis of JH, thus stimulating the development of the ovaries. Concurrently, FOXO expression is restrained, leading to the suppression of fat accumulation. Conversely, during diapause, insulin signaling is suppressed, and the JH titer is down-regulated, leading to up-regulation of FOXO expression, promoting fat hypertrophy. Our results indicate that *Contig 13394* is annotated as forkhead box protein O3 (FOXO3), which is up-regulated during diapause compared with non-diapause and diapause-terminated stages (Additional file 1), whereas the FDR of *Contig 13394* was not within the limited range (≤0.05). Therefore, the specific regulation of insulin signaling and FOXO in *C. septempunctata* L*.* during diapause must be determined through additional methods, such as RNA interference (RNAi), which will be the emphasis of future work on the molecular mechanisms of ladybug diapause*.*

## Conclusions

In this study, we presented a simple method for collecting and identifying experimental insects in diapause. In addition, we performed Illumina sequencing of *C. septempunctata* L*.* in different developmental stages, followed by read cleaning, *de novo* assembly and functional annotation, which yielded 82,820 unigenes, among which 37,872 were annotated. The most closely matched species was *T. castaneum* (10,655 unigenes), which suggests a close relationship between *C. septempunctata* L. and *T. castaneum*. Furthermore, we revealed that the type and expressed quantity of genes varied among the three different developmental stages based on pairwise comparisons. A total of 443 genes of interest were identified that had higher expression levels during diapause than during the other developmental stages. Additionally, many of these candidate genes were found to be involved in metabolism, signal transduction and hormonal control of development. Finally, we validated the transcriptome results via qRT-PCR for eight specific expressed genes. The change trends of these eight genes as identified through qRT-PCR were consistent with the RNA-seq analysis results, verifying the robustness of our results. These results increase our knowledge of the molecular biology of ladybug diapause and enhance our understanding of the molecular mechanisms of diapause.

## Methods

### Insects

*Coccinella septempunctata* L. were captured in the wheat fields of the Institute of Crop Sciences of the Chinese Academy of Agricultural Sciences and cultured in a climatic cabinet. The experimental insects were reared in plastic containers in the laboratory and fed soybean aphids daily. The insects undergo imaginal diapause; therefore, in the egg, larva and pupa stages, the rearing conditions were a temperature of 24 ± 1 °C, an RH of 70 ± 10 % and a 16:8-h light:dark (L:D) photoperiod [8, 34, 56]. Non-diapause female insects were also reared under these conditions and collected after their first oviposition. Female adults in diapause were reared at 18 ± 1 °C at an RH of 70 ± 10 % and a 10:14-h (L:D) photoperiod [56]. The experimental diapause insects were collected after 30 days. Diapause-terminated adults were transferred to another climatic cabinet with the 30-day diapause insects and reared under the same conditions as the non-diapause insects. After their first oviposition, the female insects were collected and stored at −80 °C. A Chinese patent is pending for the methods for diapause regulation and the storage and maintenance of *C. septempunctata* L. [75, 76].

### RNA preparation, cDNA library construction and RNA sequencing

Total RNA was extracted using TRIzol reagent (Life Technologies, Carlsbad, CA, USA) following the manufacturer’s instructions, and the RIN was assessed to inspect RNA integrity using an Agilent Bioanalyzer 2100 (Agilent Technologies, Santa Clara, CA, USA). The qualified total RNA was further purified with an RNeasy micro kit (Qiagen, Valencia, CA, USA) and an RNase-Free DNase Set (Qiagen, Valencia, CA, USA).

After the extraction of total RNA, oligo (dT) magnetic beads were used to isolate poly (A) mRNA, which can break mRNA into short fragments when fragmentation buffer is added. Employing these short fragments as a template, first-strand cDNA synthesis was performed using random hexamer primers, after which second-strand cDNA was synthesized in reactions including buffer, dNTPs, RNase H and DNA polymerase I. End repair of the adenylated 3’ ends was conducted on the cDNA fragments, and adapters were ligated following purification with a QIAquick PCR purification kit (Qiagen, Hilden, Germany); the fragments were then eluted with EB buffer. Suitable fragments were selected via agarose gel electrophoresis and amplified through PCR to generate the cDNA library. Finally, the concentration and size of the cDNA library was determined with a Qubit 2.0 Fluorometer (Invitrogen, Carlsbad, CA, US) and an Agilent 2100 Bioanalyzer (Agilent Technologies, Santa Clara, CA, USA). After quality inspection, sequencing was performed with the Illumina HiSeq 2500 system (Illumina, San Diego, CA, USA). Three biological replicates per treatment (non-diapause, diapause, diapause-terminated) were sequenced. The base period and mass fraction resulting from high–throughput sequencing were recorded in FASTQ format. The results of the quality evaluation of the sequencing data were excellent, with bases being distributed evenly; thus, we continued our analyses. Sequencing of the different developmental stages of ladybird adults produced raw reads.

### *De novo* transcriptome assembly and functional annotation

Raw reads were transformed from the original sequencing image data through base calling. The clean reads were filtered to remove reads with adaptors, low-quality reads (ratio of greater than 20 bases, less than 50 %), and reads showing an N ratio (unknown sequences) greater than 5 % using the FastX program (version 0.0.13) [77]. A pool of reads was formed by merging nine samples of sequencing data. *De novo* transcriptome assembly of the clean reads was performed using the scaffolding contig algorithm of CLC Genomics Workbench (version: 6.0.4) [78]. The parameters of the scaffolding contig algorithm were as follows: Word-size = 45 and minimum contig length ≥300. The final unigenes were synthesized through a second assembly, which was performed with the assembly program CAP3 EST. The final unigenes were then annotated by searching against the UniProt or Nr database using the BLASTX program [79]. We performed annotation through COG (Cluster of Orthologous Group of proteins), GO (Gene Ontology) and KEGG (Kyoto Encyclopedia of Genes and Genomes) mapping. The GO and KEGG classifications were performed using BLASTX and KAAS [80]. The expectation value (E-value) was less than 1 × 10^−5^. The COG functional categories were adopted from the rpstBlastn program based on comparison with unigenes in the CCD database. Blast2GO [81] was employed for GO functional categories according to the Gene Ontology terms molecular function, cellular component and biological process.

### Differential gene expression profiling of non-diapause, diapause and post-diapause stages

For the differential gene expression analysis, we obtained the genes with differentially expressed counts in different samples and performed GO functional analysis and KEGG pathway analysis of the differentially expressed (DE) genes. The RPKM (reads per kb per million reads) method [82] was used to calculate the expression counts of the unigenes. This method can eliminate the influence of expression levels due to differences in the lengths and amounts of sequences. The multiples of differential expression levels between different samples for a gene were calculated using the RPKM method. In addition, the RPKM of each sample as an internal standard was subjected to the Fisher test using the R language. Differentially expressed genes were identified using the Fisher test with FDR correction of the significance values. The DEGs were filtered with the following requirements: FDR ≤0.05,fold change ≥2. For pathway enrichment analysis, all of the differentially expressed genes were mapped to GO and KEGG pathway terms and the significantly enriched terms were filtered. For DEGs, GO enrichment analysis can determine the key biological functions and KEGG can determine the key biochemical metabolic pathways and signal transduction pathways.

### Quantitative real-time PCR (qRT-PCR) validation

Seven up-regulated genes and 1 down-regulated gene that were expressed during diapause were validated and quantified via real-time PCR, with three biological replicates and three technical replications. The total RNA was extracted with TRIzol reagent (Life Technologies, Carlsbad, CA, USA) for the different developmental stages (non-diapause, diapause and diapause-terminated). The diapause stage was divided into 6 phases, D1, D2, D3, D4, D5 and D6, representing diapause after 10, 20, 30, 40, 50, and 60 days, respectively. Then, 1 μg of RNA was employed for first-strand cDNA synthesis using the Prime ScriptII 1st Strand cDNA Synthesis Kit (Takara, Dalian, China) according to the manufacturer’s protocol. Real-time PCR was conducted using SYBR FAST qPCR Kit Master Mix (2×) Universal (KAPA Biosystems, Woburn, MA, USA) under the following conditions: 95 °C for 5 min, followed by 40 cycles of 95 °C for 30 s and 60 °C for 20 s. The melting curve was analyzed from 60 °C to 95 °C to detect nonspecific product amplification. The 18S-RNA gene of *C. septempunctata* L. was used as an internal gene. The primers used for real-time PCR are listed in Additional file 6. All of the data obtained through qRT-PCR were analyzed via the 2^-ΔΔCT^ method.

## Availability of supporting data

All Illumina data used in the present study have been deposited in NCBI’s Gene Expression Omnibus (GEO) under accession number GSE75645.

## Additional files

Additional file 1:
**S1-Unigene expression.** (XLSX 4.86 mb) Additional file 2:
**S2-Gene Ontology classification.** (PDF 6.16 kb) Additional file 3:
**S3-Significantly enriched KEGG pathways in D vs. ND and DT vs. D.** (XLSX 16.6 kb) Additional file 4:
**S4-Differentially expressed genes involved in fatty acid biosynthesis and cell cycle pathways.** (XLSX 11.8 kb) Additional file 5:
**S5-Diapause-associated genes of interest.** (XLSX 12.0 kb) Additional file 6:
**S6-The primer of qRT-PCR.** (XLSX 10.3 kb)

## Abbreviations

BLAST: Basic local alignment search tool
COG: Clusters of orthologous groups of proteins
GO: Gene ontology
KEGG: Kyoto encyclopedia of genes and genomes
ND: Non-diapause
D: Diapause
DT: Diapause-terminated
DEGs: Differentially expressed genes
qRT-PCR: Quantitative real-time polymerase chain reaction
RNA-Seq: RNA sequencing
FOXO: Forkhead box protein o
L: D: Light: dark
NGS: Next-generation sequencing technology
DG: Diapause-associated gene
ORF: Open reading frame
CDD: Conserved domain database
KAAS: KEGG automatic annotation server
FDR: False discovery rate
RPKM: Reads per kb per million reads
TLR: Toll-like receptor
FAH: Fumarylacetoacetase
JH: Juvenile hormone
JHE: Juvenile hormone esterase
SDH: Sorbitol dehydrogenase
GP: Glycogen phosphorylase
SP: Serine protease
JHEH: Juvenile hormone epoxide hydrolase
TRE: Trehalase
PCNA: Proliferating cell nuclear antigen
EST: Expressed sequence tag
