# Supplementary figures and images for: De novo transcriptome sequencing and analysis of Coccinella septempunctata L. in non-diapause, diapause and diapause-terminated states to identify diapause-associated genes
Source: BMC Genomics. 2015 Dec 21;16:1086. doi: 10.1186/s12864-015-2309-3 (PMC4687109; doi:10.1186/s12864-015-2309-3)

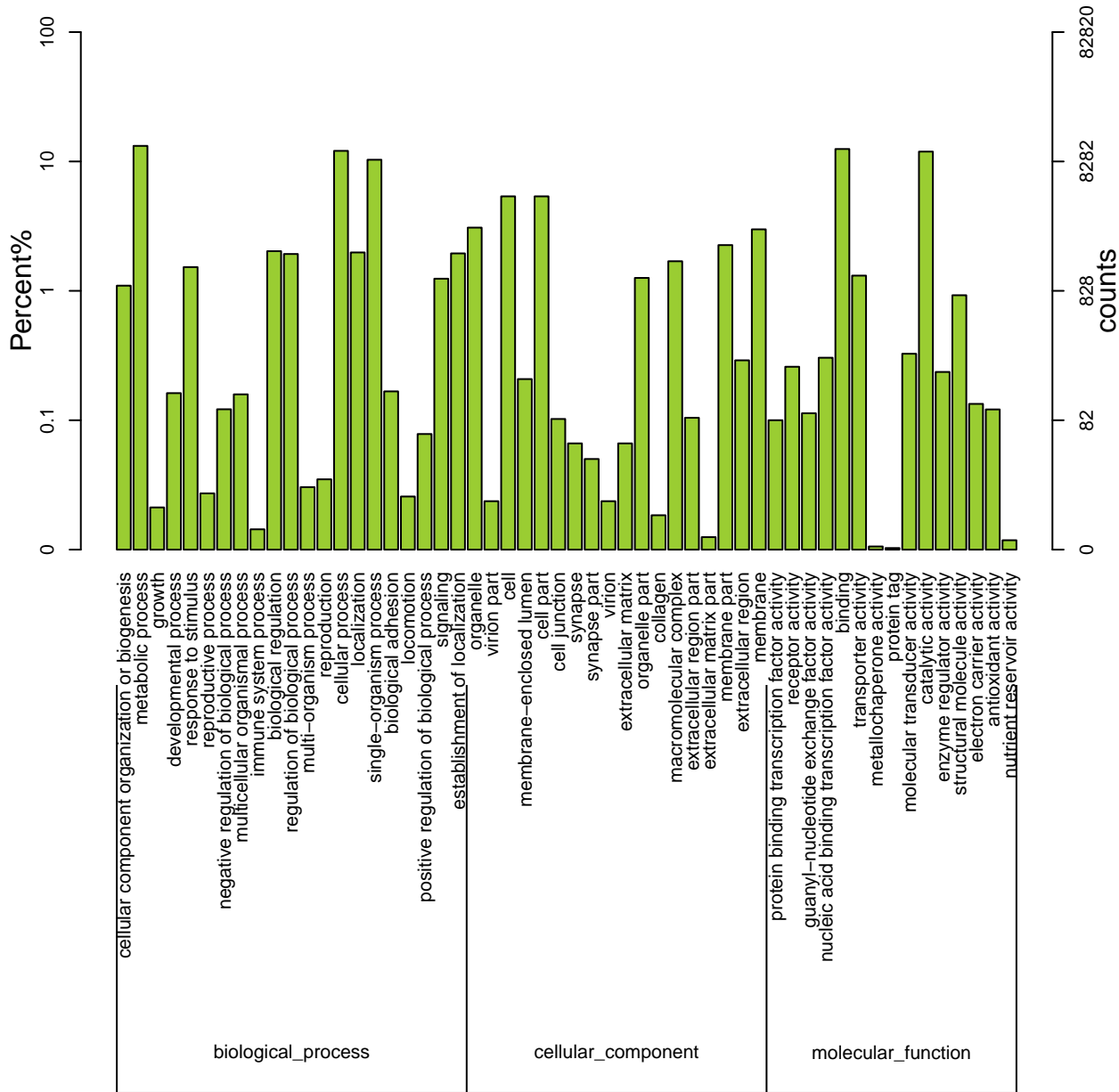

Supplement: Additional file 2: — S2-Gene Ontology classification. (PDF 6.16 kb) [file 12864_2015_2309_MOESM2_ESM.pdf]
